# Optical Properties of Conical Quantum Dot: Exciton-Related Raman Scattering, Interband Absorption and Photoluminescence

**DOI:** 10.3390/nano13081393

**Published:** 2023-04-18

**Authors:** Sargis P. Gavalajyan, Grigor A. Mantashian, Gor Ts. Kharatyan, Hayk A. Sarkisyan, Paytsar A. Mantashyan, Sotirios Baskoutas, David B. Hayrapetyan

**Affiliations:** 1 Department of General Physics and Quantum Nanostructures, Russian-Armenian University, 123 Hovsep Emin Str., Yerevan 0051, Armeniagrigor.mantashyan@rau.am (G.A.M.); gor.kharatyan@rau.am (G.T.K.); hayk.sarkisyan@rau.am (H.A.S.); david.hayrapetyan@rau.am (D.B.H.); 2 Institute of Chemical Physics after A.B. Nalbandyan of NAS RA, 5/2 Paruyr Sevak St., Yerevan 0014, Armenia; 3 Department of Materials Science, University of Patras, 265 04 Patras, Greece

**Keywords:** conical quantum dot, exciton, Raman scattering, interband absorption, photoluminescence

## Abstract

The current work used the effective mass approximation conjoined with the finite element method to study the exciton states in a conical GaAs quantum dot. In particular, the dependence of the exciton energy on the geometrical parameters of a conical quantum dot has been studied. Once the one-particle eigenvalue equations have been solved, both for electrons and holes, the available information on energies and wave functions is used as input to calculate exciton energy and the effective band gap of the system. The lifetime of an exciton in a conical quantum dot has been estimated and shown to be in the range of nanoseconds. In addition, exciton-related Raman scattering, interband light absorption and photoluminescence in conical GaAs quantum dots have been calculated. It has been shown that with a decrease in the size of the quantum dot, the absorption peak has a blue shift, which is more pronounced for quantum dots of smaller sizes. Furthermore, the interband optical absorption and photoluminescence spectra have been revealed for different sizes of GaAs quantum dot.

## 1. Introduction

Researchers have recently concentrated on the physical characteristics of low-dimensional semiconductor structures such as zero-dimensional quantum dots (QDs) and two-dimensional quantum wells. Because of their potential for device applications in photodetectors, infrared laser amplifiers, electro-optical modulators, etc., it is possible to retrace the development of works on linear and nonlinear optical properties, particularly interband absorption [1,2,3,4,5,6] and refractive index change [7]. In the last decades, several writers have conducted experimental and theoretical studies on the nonlinear optical properties of QDs [8,9,10]. It is commonly recognized that a QDs form can cause certain physical behaviors. Therefore, numerous investigations have been carried out to examine the optoelectronic characteristics linked to various QD geometries, including spherical [11], cylindrical [12], ring [13], ellipsoidal [14,15], conical [16], etc.

Although QDs with trivial geometry, like spherical and cylindrical, can be theoretically investigated analytically, the QDs with complicated structures can be investigated in approximation or numerical methods. For example, spheroidal or ellipsoidal QDs can be investigated with the help of geometrical adiabatic approximation [17,18,19].

In those works [20,21], with the help of the adiabatic approximation method, authors could calculate direct light absorption in coated ellipsoidal and spheroidal QDs. The geometrical adiabatic approximation method can also be applied to the conical quantum dot (CQD). In [16], within the framework of the adiabatic approximation, the energy states of an electron and the direct absorption of light in a conical quantum dot in an external magnetic field were studied.

Parallel to approximation methods, different numerical methods have been developed and applied to investigate QDs. For example, such as the strong confinement approximation [22,23], the variational approach [24,25,26,27,28,29,30,31] and the perturbation theory [32].

For example, in [30], authors theoretically studied the donor-impurity-related optical absorption, relative refractive index changes, and Raman scattering in GaAs cone-like quantum dots using the variational approach.

Xie [31] examined the optical characteristics of a GaAs spherical QD with parabolic confinement potential for two cases with donor and acceptor impurities in the presence of a static electric field within the framework of the variational approach, demonstrating that the optical characteristics of such a system are significantly influenced by the degree of quantum confinement, the strength of the applied electric field, as well as by the impurity properties. Bose et al. [32] employed a perturbation method to calculate the donor states associated with a few higher-lying levels, including the lowest one in a spherical QD.

One of the powerful numerical methods for calculating the electronic structure of QDs is the finite element method (FEM), a method for solving equations that approximates continuous quantities as a collection of discrete quantities at regular intervals to form a grid or mesh [33,34,35,36]. FEM is one of the most effective numerical tools that may be utilized for this aim. This approach allows us to solve the eigenvalue problem quickly and efficiently for QDs with complex morphological and potential profiles without needing a supercomputer or computing cluster. The authors of [33] utilized FEM to calculate the first twenty-six probability densities and energy values for the following GaAs structures: rectangular, spherical, cylindrical, ellipsoidal, spheroidal, and conical QDs, as well as quantum rings, nanotadpoles, and nanostars. The authors’ numerical calculations were compared to the exact analytical solutions, and a good divergence was found. Thereby, the FEM make it possible to calculate the energy spectra for charge carrier in the semiconductor QD with any shape and size [36] where authors implemented FEM to investigate the electronic state of shallow hydrogenic impurities in spherical GaAs QD. In the current manuscript, the FEM has been applied for the investigation of optical properties of exciton in conical QD.

The determination of excitons in QDs has important significance for the realistic modeling of these nanostructures. Apart from being of academic interest, these systems resemble the hydrogen atom and have practical relevance, particularly in heterostructure lasers where the radiation is affected by electron-hole recombination.

Notably, the binding energy of such a system necessitates a further definition. It is defined by the difference between the energy of the exciton and the energy of a one-particle electron (hole). The exitonic phenomenon has an instantaneous impact on the physical characteristics of the QD, particularly the optical characteristics [37,38,39]. Since photoluminescence (PL) spectroscopy is a nondestructive characterization tool frequently utilised to identify shallow-level and occasionally deep-level impurities that produce radiative recombination, it is crucial to investigate the PL properties of various QDs. An effective method for examining the optical characteristics of fluorophores is PL spectroscopy. [40,41,42]. The analysis of optical characteristics, specifically the PL spectra of various QD kinds, is thus a pertinent and cutting-edge problem in semiconductor nanophysics.

Finally, Raman spectroscopy, which is utilized in a variety of scientific activities, is one of the efficient techniques for examining QDs. For example, in solid-state physics, the Raman scattering peak’s position, shape, and intensity can reveal information about a material’s electronic structure and lattice dynamics. On the other hand, for a deeper knowledge of semiconductor nanostructures, the Raman differential effective cross-section computation continues to be a pretty intriguing and vital subject [43,44,45,46,47,48,49,50,51]. In [45], the authors studied the Raman scattering process connected to an exciton contained by a quantum dot using the matrix diagonalization approach within the effective-mass approximation.

In general, the optical properties of QDs can be useful for designing novel photonic devices in various ways. For instance, by understanding the size and composition-dependent absorption and photoluminescence spectra of QDs, researchers can engineer the QDs to achieve specific optical properties, such as narrow emission line widths or high photoluminescence quantum yields [47,48,49,50]. Furthermore, these properties are crucial for designing high-performance photonic devices like lasers, displays, and sensors [51,52,53,54].

Moreover, by engineering the band structure of QDs, it is possible to enhance light absorption and charge separation in solar cells. As a result, QDs with a graded bandgap can efficiently harvest a broad range of solar radiation, increasing the efficiency of solar cells [55,56,57].

Therefore, the obtained results on the optical properties of conical QDs provide valuable insights for designing and optimizing photonic devices with enhanced performance and functionality, making them useful for a wide range of applications, including solar cells, lighting, displays, and sensing.

The current work aims to quantify the exciton energy in a CQD with infinite confinement potential. The paper is structured as follows: we will use perturbation theory to apply to solve the problem, then we determine the excitonic energy by numerical methods, particularly the FEM, calculate exciton lifetime, absorption, photoluminescence, and Raman spectroscopy, and finally, we discuss our results and present our conclusion.

## 2. Materials and Methods

### 2.1. Exciton States in Conical Quantum Dots

As a first step, we will consider exciton states in conical QDs and solve the problem in which the cone’s height (H) and radius (R) are comparative values. Unfortunately, this means that the problem cannot be solved analytically, and we must apply either approximate methods or numerical methods to solve the problem. Next, we will consider the case of conical QD with impenetrable walls when the potential of the particle inside the QD is zero and infinite outside:

(1) $$U_{conf}\left({\vec{r}}_{e\left(h\right)}\right)=\left\{\begin{array}{c} 0,\;\mathrm{particle}\;\in \Delta ,\\ \propto ,\;\mathrm{particle}\;\notin \Delta . \end{array}\right.$$

**Figure 1:**
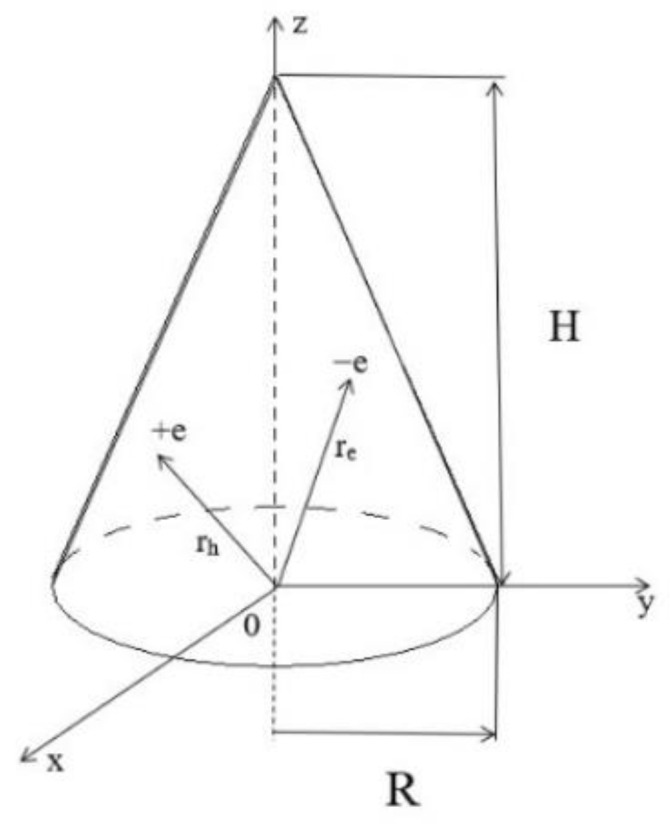
Schematic figure of the conical quantum dot.

The schematic plot of the conical QD is presented in Figure 1. Where the geometrical parameters of the conical QD have been depicted.

In this case, the Hamiltonian of the exciton system in Cartesian coordinates can be written as follows:

(2) $$\hat{H}=-\frac{\hbar^{2}}{2m_{e}^{*}}\Delta_{e}-\frac{\hbar^{2}}{2m_{h}^{*}}\Delta_{h}+U_{conf}\left({\vec{r}}_{e}\right)+U_{conf}\left({\vec{r}}_{h}\right)+V_{eh}\left({\vec{r}}_{e},{\vec{r}}_{h}\right)$$

where $m_{e}^{*}$ and $m_{h}^{*}$ are the effective masses of the electron and hole, respectively, $V_{eh}\left({\vec{r}}_{e},{\vec{r}}_{h}\right)=-\frac{e^{2}}{\varepsilon \left|{\vec{r}}_{e}-{\vec{r}}_{h}\right|}$ is the Coulomb interaction potential between electron and hole, ε is the dielectric permittivity of the GaAs, $U_{conf}\left({\vec{r}}_{e}\right)$ and $U_{conf}\left({\vec{r}}_{h}\right)$ are, respectively, confinement potentials for the electron and hole.

The problem of finding exciton states can be solved within the variation principle. In the case of an intermediate-size quantization regime, the wave function of the exciton can be presented in the form:

(3) $$\Psi_{exc}({\vec{r}}_{e},{\vec{r}}_{h})=\psi_{e}\left({\vec{r}}_{e}\right)\psi_{h}\left({\vec{r}}_{h}\right)Exp\left\{-\lambda \left|{\vec{r}}_{e}-{\vec{r}}_{h}\right|\right\}$$

Here λ is the variation parameter, while $\psi_{e}\left({\vec{r}}_{e}\right)$ and $\psi_{h}\left({\vec{r}}_{h}\right)$ are single-particle wave functions for the electron and hole, respectively. Single particle problems can be solved in the framework of the FEM, which allows us to solve the problem in the general case when not only the geometrical sizes are comparable but also for the intermediate size quantization regime. In this case, the size quantization energy and the Coulomb interaction between an electron and a hole contribute to the system’s overall energy. Thus, none of these terms can be neglected in the Hamiltonian (2) of the system. In this case, the exciton energy in the framework of the variation theory can be calculated by the minimization of the following equation:

(4) $$E_{exc}=\langle \Psi_{exc}({\vec{r}}_{e},{\vec{r}}_{h})\left|\hat{H}\right|\Psi_{exc}({\vec{r}}_{e},{\vec{r}}_{h})\rangle$$

The dependence of the exciton energy on the geometrical parameters of the conical QD will be discussed later in the results paragraph.

Based on the calculated exciton energy, we can easily determine the binding energy of the exciton, which present the difference between the energies of the separate electron and hole and exciton energy:

(5) $$E_{bind}=\left(E_{e}+E_{h}\right)-E_{exc}$$

Before investigating the exciton-related optical properties of conical QDs, it is logical to estimate the exciton lifetime in the system. Therefore, we employ the formula obtained in [58] to calculate the radiative lifetime of exciton states in QDs:

(6) $$\tau_{exc}=\frac{2\pi \varepsilon_{0}m_{e}c^{3}\hbar^{2}}{\sqrt{\varepsilon }e^{2}E_{exc}^{2}f}$$

Here f is the strength of the oscillator, which can be calculated based on the following equation:

(7) $$f=\frac{E_{P}}{E_{exc}}{\left|\int \Psi_{exc}\left({\vec{r}}_{e},{\vec{r}}_{h}\right)d{\vec{r}}_{e}d{\vec{r}}_{h}\right|}^{2}$$

where c is the light velocity and $E_{p}$ is the energy of Kane. The presented equation for the lifetime applies only to the materials with parabolic dispersion relation. Hence, the application for the materials such as PbS, PbTe, etc., that have extremely accentuated non-parabolic dispersion relation is limited. Another limitation is that this lifetime only addresses the radiative recombination neglecting the non-radiative pathways, such as Auger recombination, phonon scattering etc.

### 2.2. Optical Properties

The interband absorption spectra of the incident light, photoluminescence, and Raman shift of conical QD are investigated in this section. The spectral line broadening is accounted for by the light absorption coefficient, which takes the following form:

(8) $$\alpha \left(\hbar \omega ,R,H\right)=\alpha_{0}{\sum_{n_{e},n_{h}}\left|\int \Psi_{exc}\left({\vec{r}}_{e},{\vec{r}}_{h}\right)d{\vec{r}}_{e}d{\vec{r}}_{h}\right|}^{2}\frac{\Gamma_{0}}{{\left(\hbar \omega -E_{exc}\right)}^{2}+\Gamma_{0}^{2}}$$

where $E_{g}$ is the band gap of the semiconductor, $\alpha_{0}$ is a value proportional to the square of the matrix element of the dipole moment, taken from the Bloch functions; $\Gamma_{0}$—Lorentzian parameter [59,60]. The parameter $\Gamma_{0}$ takes into account the homogeneous broadening of the absorption lines.

Based on the calculated absorption spectra, we can determine the photoluminescence spectra for conical QD by using the Rusbroek-Shockley relation [61,62]:

(9) $$R\left(\hbar \omega ,R,H\right)=R_{0}\hbar \omega \;\alpha \left(\hbar \omega ,R,H\right)\frac{f_{c}\left(1-f_{v}\right)}{f_{v}-f_{c}}$$

where $R_{0}$ is proportional to the square of the matrix element of the dipole moment, taken on the Bloch functions, $f_{c}$ and $1-f_{v}$ are the probabilities of filling the states of the conduction band and the emptiness of the states of the valence band, respectively.

According to the third-order perturbation theory, the expression produced for the computation of the exciton-related Stokes-Raman scattering DCS in a volume V, per unit solid angle dΩ, takes the following form:

(10) $$\frac{d^{2}}{d\Omega d\omega_{s}}=\frac{V^{2}\omega_{s}^{2}\eta (\omega_{s})}{8\pi^{3}c^{4}\eta (\omega_{l})}W(\omega_{s},\vec{e_{s}})$$

Here, the incident light frequency is $\omega_{l}$, the scattered light frequency is $\omega_{s}$, and the refractive indices of the incident and scattered light, re $\eta (\omega_{s})$ and $\eta (\omega_{l})$. The polarization vector is denoted by $e_{s}$ [63,64,65]. The transition rate, denoted by $W(\omega_{s},\vec{e_{s}})$, can be determined using the following equation:

(11) $$W(\omega_{s},\vec{e_{s}})=\frac{2\pi }{\hbar }{\sum_{f}\left|M_{e}+M_{h}\right|}^{2}\delta (E_{f}-E_{i})$$

where M and $\delta (E_{f}-E_{i})$ are defined by the following expressions:

(12) $$M_{j}=\sum_{a}\frac{\langle f|H_{js}|a\rangle \langle a|H_{js}|i\rangle }{E_{i}-E_{a}-i\Gamma_{a}},\;\;\;j=e,h$$

(13) $$\delta (E_{f}-E_{i})=\frac{\Gamma_{f}}{\pi \{{\left(E_{f}-E_{i}\right)}^{2}+\Gamma_{f}^{2}\}}$$

Consider that the terms $|i\rangle$, $|a\rangle$, and $|f\rangle$ respectively, characterize the system’s initial, intermediate, and final states with energies $E_{i}$, $E_{a}$, $E_{f}$ and the lifetime width is $\Gamma_{f}$. The interaction between the incident and secondary radiation fields can be expressed by the Hamiltonian operators in the dipole approximation:

(14) $$\begin{array}{l} {\hat{H}}_{ji}=\frac{\left|e\right|}{m_{e}}\sqrt{\frac{2\pi \hbar }{V\omega_{l}}}({\vec{e}}_{jl}\cdot {\vec{p}}_{j}),\;\;\;\;{\vec{p}}_{j}=-i\hbar {\vec{\nabla }}_{j}\\ {\hat{H}}_{js}=\frac{\left|e\right|}{m_{j}^{*}}\sqrt{\frac{2\pi \hbar }{V\omega_{s}}}({\vec{e}}_{js}\cdot {\vec{p}}_{j}) \end{array}$$

where, ${\vec{e}}_{jl}$ and ${\vec{e}}_{js}$ are the unit polarization vectors for the incident and secondary radiations. The matrix elements of DCS have been calculated using numerical values for the energy spectra and wave functions obtained in Section 1.

## 3. Results and Discussion

Let’s proceed with the analysis of the outcomes. The material parameters are taken from [66]. For various-sized QDs, the homogeneous broadening linewidth is assumed to be $\Gamma_{0}=5(\mathrm{meV})$ [66,67]. For the lifetime-width $\Gamma_{f}$, we will also take the same value as for the homogeneous broadening linewidth.

**Figure 2:**
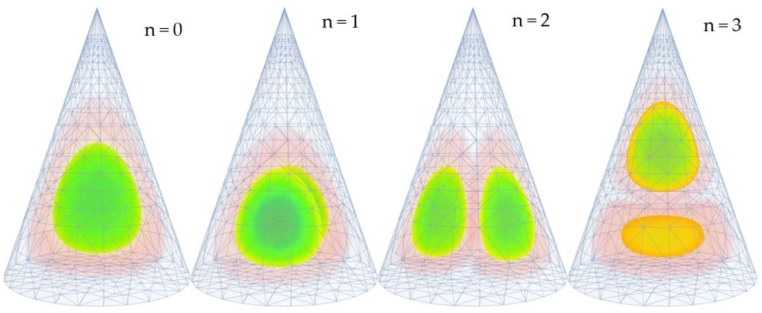
Probability distribution of an electron (hole) over conical QD for the first four states. The color scale in the figure is from green (high density) to red (low density).

The probability distributions of an electron (hole) over conical QDs for different quantum levels are shown in Figure 2. We should note that n it is a numbering of the states and does not present a quantum number. The first figure presents the ground state of the electron in conical QD. As we can see from the figure, the electron is mainly localized in the center of the cone and has a slight asymmetry because of the influence of the cone walls. The other three cases present the first three excited states, which correspond to the p states of the spherical QD.

In contrast to the spherical case, the degeneracy order of these states in conical QD is two. It can be seen clearly from the probability distribution of the electron in radial directions and from the corresponding energy values. The fourth state has elongation alongside the axial direction, and its energy is higher than the energies of the degenerated states.

**Figure 3:**
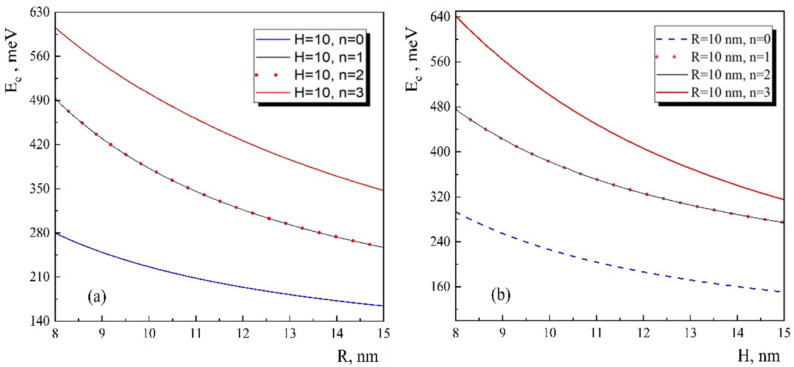
The dependences of the electron energy on the (**a**) radius of the conical QD and (**b**) height of the conical QD for the first four levels.

The abovementioned is demonstrated in Figure 3, where the energies of the electron have been presented as a function of the radius and height of the conical QD for the first four levels. As can be seen from the figure, in all cases, the energy levels decrease with the increase of geometrical parameters of the conical QD. This is due to the weaknesses of the size quantization effect. Furthermore, the second and the third states have degeneracy since they correspond to the states that have π/2 rotation in contrast to the conical QD axes.

**Figure 4:**
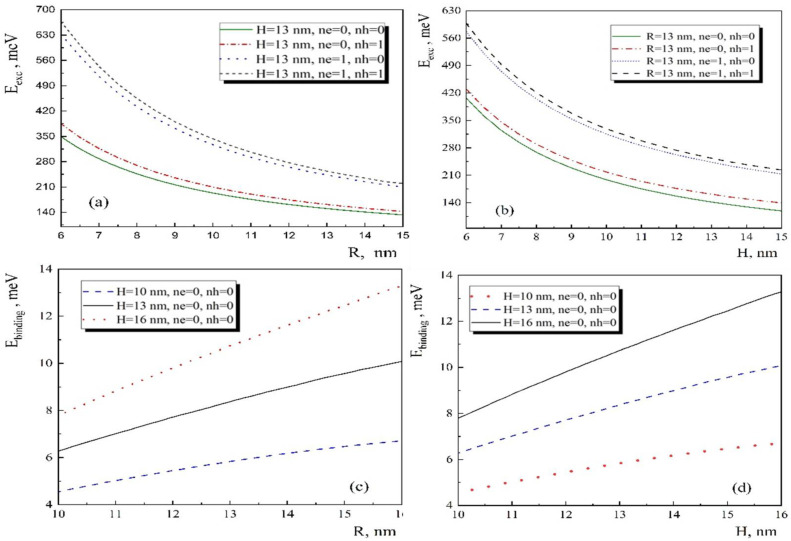
The exciton energy and binding energy depend on the geometrical parameters of the conical QD. (**a**,**b**) dependence of the exciton energy on radius and height for the first four states, respectively. (**c**,**d**) dependence of the binding energy on the radius and height for the ground state, respectively.

As we mentioned in the previous section, in the framework of the variation theory, we can calculate the exciton energy and binding energy based on the single particle wave functions using Equations (3) and (4). The obtained results are presented in Figure 4. The dependences of the exciton energies for the first four levels have been illustrated on the cone radius (see Figure 4a,c) and the cone height (see Figure 4b,d), respectively. In both cases, the exciton energy decreases with the increase of geometrical parameter since the size quantization effect become weaker. In contrast to the exciton energy, the exciton binding energy, calculated by Equation (5), increases with the increase of geometrical parameters. Let us note that the binding energy has a positive value, which means that the exciton is a stable quasiparticle in conical QD.

Based on the calculated exciton energies for the conical QD, we can estimate the exciton radiative lifetime, which is determined by Equation (6). The calculated values of the radiative lifetime depend on the different geometrical parameter values presented in Table 1. Note that when estimating the radiative lifetime, we neglected the interaction of the exciton with phonons. The characteristic times are in the range of nanoseconds. As seen from Table 1, with an increase in the QD size, the radiative lifetime increases accordingly. The increase in the radiative lifetime can be explained as follows: with an increase in the QD size, the exciton energy and the overlap integral in the oscillator strength formula decrease since the size quantization effect weakens.

**Figure 5:**
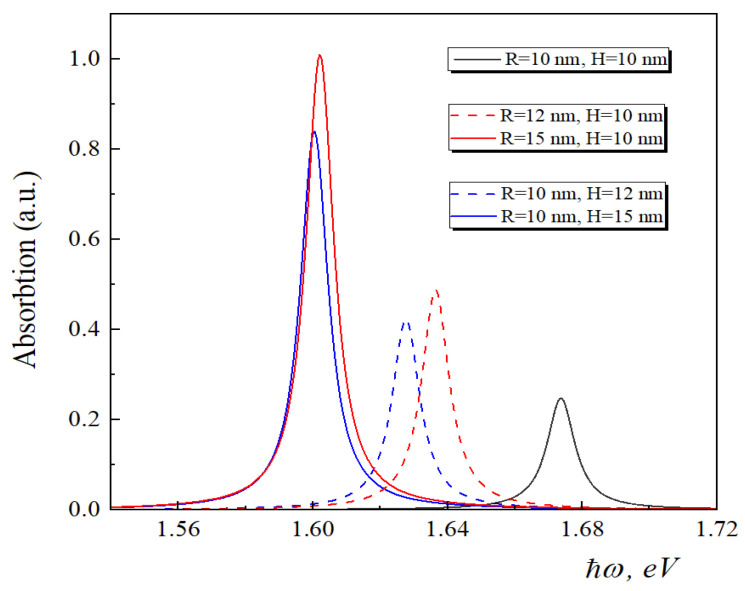
Dependence of the absorption lines on the energy of the incident light for various sizes of conical QD for the quantum transition from the ground state to the first excited state.

In Figure 5, the dependence of the absorption lines on the energy of incident light for different sizes of GaAs conical QD has been presented for the quantum transition from the ground state to the first excited state. As we can see from the figure, with the decrease in the QD size, the absorption peak has a blue shift. The shift has an asymmetric character. Moreover, for the smaller sizes, the shift is more pronounced. This is the expression of the size quantization: the QDs with smaller sizes have higher exciton energy. We present two sets of absorption lines: in the first set (red curves), the height of the conical QD is fixed, and in the second set (blue curves), the radius of the base of the conical QD is fixed. The black curve in the figure corresponds to the common case for the two sets. It is visible from the figure that the change in radius and height affect differently on the position and the intensity of the absorption line. The change in height brings more shifts in the position of the absorption peak than the shift in radius.

**Figure 6:**
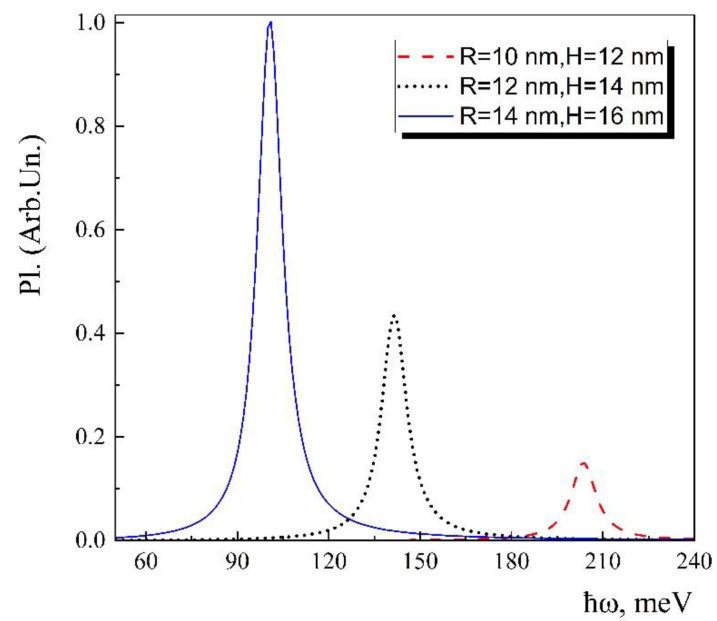
Dependence of the light energy of the PL spectra for different conical QD sizes at 4.2 K temperature.

We can calculate PL spectra based on the absorption coefficient considering the Rusbroek-Shockley relation (9). The dependence of the PL spectra on the incident light energy is shown in Figure 6 for the three sets of geometrical parameters. Similar behaviors for the dependence of the PL on the geometrical parameters of the conical QD to the absorption case are also observed in this case. Note that the difference is only in the intensities of the PL peaks, which are modified in accordance with the Rusbroek-Shockley relation. The decrease in the intensities of the PL peaks in contrast to the absorption peaks is due to the exponential dependence of the PL on the temperature. Such exponential dependence is caused by the $\frac{f_{c}\left(1-f_{v}\right)}{f_{v}-f_{c}}$ factor, which has Boltzmann form for the low temperatures [61].

**Figure 7:**
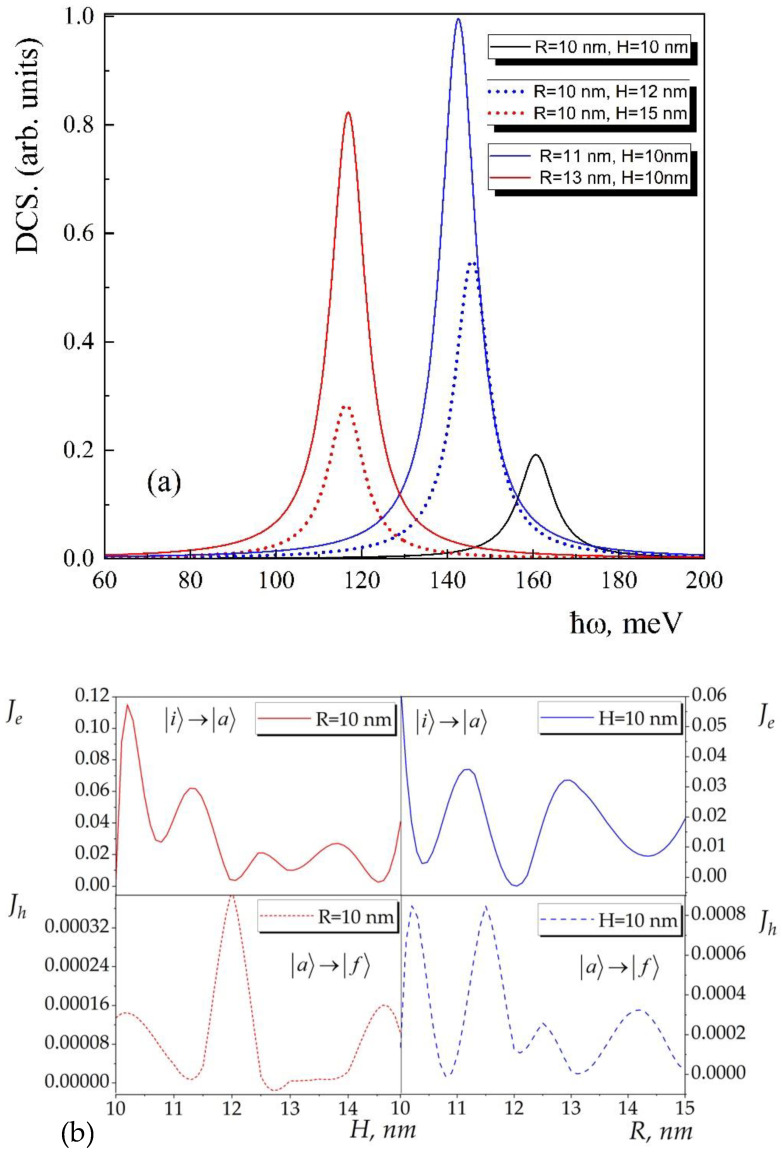
Exciton-related Raman DCS as a function of secondary-radiation photon energy for different sizes of GaAs conical QD (**a**) and overlap integrals as a function of the geometrical parameters H and R for the transitions $|i\rangle \to |a\rangle$ and $|a\rangle \to |f\rangle$ (**b**).

Finally, consideration has been given to the exciton-related Raman DCS for various GaAs conical QD sizes as a function of secondary-radiation photon energy has been considered. As with any property requiring inter-level transition, the DCS strongly depends on the inter-level energy difference. Considering that the energy levels and their distance depend on the geometrical parameters of the dot, the peak position and oscillatory strength are also highly dependent on the geometry. Equation (10) is used to get the DCS of Stokes Raman scattering for a three-level system for a single GaAs QD. We take Raman excitations from the exciton’s ground state, $|i\rangle$, which has been chosen as the beginning state. The energy of an incident photon is $\hbar \omega_{l}$, but the energy of light that is scattered is $\hbar \omega_{s}$. First, there is a transition from the ground state, which has energy $E_{exc}^{i}$, to the intermediate state $|a\rangle$, which has energy $E_{exc}^{a}$, and the second is the excited state. The energy of the secondary radiation $\hbar \omega_{s}$ comes from the last transition indicated above. All excited states beginning with the second can be regarded as intermediate $|a\rangle$. To simplify it, we’ll discuss the scenario when the intermediate state is the second excited state. This approach justifies the comparatively minor contribution of the transitions from the upper excited states. Figure 7 shows the three-level transition processes $\left(1\to 3\to 2\right)$ together with the Raman DCS outcomes. The final and intermediate states’ lifetime widths are determined as follows: $\Gamma_{f}=\Gamma_{a}=5\mathrm{meV}$ [67]. It should be noted that because the exciton phonon interaction is considered, these empirically measured lifetime widths deviate from radiative lifetime.

Figure 7a, the dependence of the exciton-related Raman DCS as a function of secondary-radiation photon energy for different sizes of GaAs conical QDhas. As we can see from the figure, there are two sets of peaks, one of which corresponds to the case when we fixed the height H of the conical QD and changed the radius R of the bases and the second one to the opposite case. The peak position of the Raman DCS is conditioned by the energy difference between the ground state $|i\rangle$ and the final state $|f\rangle$, while the peak intensity is defined by the overlap integrals $J_{e}$ and $J_{h}$ which are included in the matrix elements $M_{e}$ and $M_{h}$. These overlap integrals have interesting behavior as a function of the geometrical parameters H and R. They are presented in Figure 7b for the corresponding transitions $|i\rangle \to |a\rangle$ and $|a\rangle \to |f\rangle$. These overlap integrals defined selection rules for the corresponding transitions. Thus, depending on the H/R ratio, transitions can occur or can disappear. Overlap integral value dependence on the height or radius of the conical QD has approximately periodical character. Herewith the maximum value of the overlap integral for the $|i\rangle \to |a\rangle$ transition is a few orders higher than for the $|a\rangle \to |f\rangle$ transition.

## 4. Conclusions

This study investigated the excitonic properties of conical quantum dots (QDs) with impenetrable walls. First, the probability distributions of electrons and holes over conical QDs for different quantum levels were analyzed. It was shown that the energy levels decrease with the increase of geometrical parameters of the conical QD. Second, the exciton and binding energy were calculated based on the single-particle wave functions. It was found that the exciton energy decreases while the exciton binding energy increases with the increase of geometrical parameters. Third, the radiative lifetime was estimated based on the calculated exciton energies for the conical QD. It was observed that with an increase in the QD size, the radiative lifetime increases accordingly. The dependence of the absorption lines and photoluminescence spectra on the incident light energy for different sizes of GaAs conical QD were also analyzed. Finally, the exciton-related Raman DCS for various GaAs conical QD sizes was investigated as a function of secondary-radiation photon energy.

The obtained results provide a better understanding of the optical properties of conical QDs and can be useful for designing novel photonic devices. For example, they can be used to engineer the size and composition of QDs to achieve specific optical properties, such as narrow emission line widths or high photoluminescence quantum yields. These properties are important for designing high-performance photonic devices such as lasers, displays, and sensors. In another case, by engineering the band structure of the quantum dots, it is possible to enhance light absorption and charge separation in solar cells.

## Figures and Tables

**Table 1:** Values of the radiative lifetime for the different values of radius and height of the conical QD.

R, nm	H, nm	$\tau_{exc},\;ns$
8	12	0.98
	14	1.09
	16	1.18
10	12	1.32
	14	1.49
	16	1.651
12	12	1.656
	14	1.91
	16	2.14

## Data Availability

Not applicable.
